# The Post-Stroke Checklist: longitudinal use in routine clinical practice during first year after stroke

**DOI:** 10.1186/s12872-024-04239-6

**Published:** 2024-10-29

**Authors:** Kristina Månsson, Martin Söderholm, Ida Berhin, Hélène Pessah-Rasmussen, Teresa Ullberg

**Affiliations:** 1 Department of Neurology, Skåne University Hospital, Jan Waldenströms gata 15, Malmö, 205 02 Sweden; 2 Department of Clinical Sciences Lund, Neurology, Lund University, Jan Waldenströms gata 15, Lund, 205 02 Sweden; 3 Department of Clinical Sciences Malmö, Epidemiology, Lund University, Jan Waldenströms gata 15, Lund, 205 02 Sweden; 4 Department of Neurology, Rehabilitation Medicine, Memory Clinic and Geriatrics, Skåne University Hospital, Malmö, Sweden; 5 Department of Clinical Sciences Lund, Rehabilitation Medicine, Lund University, Lund, Sweden

**Keywords:** Stroke, Stroke prevention, Stroke rehabilitation, Outcome, Quality of care, Follow-up, Modifiable stroke risk factors, Complications

## Abstract

**Background:**

Few studies describe the use of the Post-Stroke Checklist (PSC) as a tool for longitudinal stroke follow-up in clinical practice. We mapped the prevalence of stroke-related health problems and targeted interventions at 3 and 12 months post-stroke by using the PSC.

**Methods:**

Patients with acute stroke discharged home in 2018–2019 at Skåne University Hospital, Sweden, were invited to participate in a comprehensive nurse-led follow-up based on a 14-item PSC 3 and 12 months post-stroke. We measured time consumption, screened for stroke-related health problems, compared the findings, and recorded targeted healthcare interventions. Problems at 12 months were grouped into new, persistent, or none compared to the 3-month evaluation.

**Results:**

Of 200 consecutively included patients, 146 (77%) completed both the 3- and 12-month follow-ups. At 12-month follow-up, 36% of patients reported no stroke-related health problems, 24% reported persistent problems, and 40% reported new problems since the 3-month evaluation. New problems at 12 months were most common within the domains: *secondary prevention* (23%) and *life after stroke* (10%). Stroke recurrence rate was 7.5%, 43% had high blood pressure, and few smokers had quit smoking. At 12 months, 53% received at least one new healthcare intervention, compared to 84% at 3 months.

**Conclusions:**

Stroke-related health problems decreased beyond 3 months but were still present in two-thirds of patients at 1 year. This emphasizes the relevance of continuous structured follow-up using the PCS. However, the follow-up alone was insufficient to adequately achieve treatment targets for secondary prevention, which require intensified focus.

**Trial registration:**

ClinicalTrials.gov ID NCT04295226, (04/03/2020)

**Supplementary Information:**

The online version contains supplementary material available at 10.1186/s12872-024-04239-6.

## Background

As a result of a growing aging population and improved stroke survival, the absolute number of stroke survivors is predicted to increase [1–3]. Developing resource-effective and sustainable models for the management and care of stroke survivors in a long-term setting is highly relevant.

The Stroke Action Plan for Europe, a European collaborative project with an overall aim to reduce the burden of stroke by improving stroke care, has introduced *Life after stroke* as a new domain important to address in stroke survivors. The Stroke Action Plan provides a recommendation that stroke survivors should be offered follow-up at 3–6 months post-stroke based on the Post-Stroke Checklist (PSC). (4–5) This is in line with recommendations in the Swedish national guidelines for stroke care [6]. Still, structured stroke follow-up is not yet fully established on a national or even regional basis, indicating a major gap between guideline recommendations and long-term management of stroke survivors. According to SSNAPP (The Sentinel Stroke National Audit Programme), which measures quality of stroke care in the U.K, only 35% of patients applicable for follow-up completed a 6-month follow-up [7]. In Sweden, there are no reliable data on the proportion of stroke survivors that receive follow-up visits, confirming the gap. Unfortunately, the transition between in- and outpatient care is an area in which problems often occur. Individuals who are discharged home risk inconsistencies in follow-up care and therefore are an important group to study, especially having in mind that this stroke population represents 75% of all stroke patients in Sweden [8]. 

A systematic review from 2021 summarizes the current knowledge on the organization of post-stroke care by targeting the several crucial aspects (neurological deficit, any post-stroke complications, inadequately treated risk factors, and unmet psychosocial needs) affecting the long-term impairments and quality of life of stroke survivors [9]. The STROKE-CARD care trial showed that a comprehensive post-stroke care program handling the multifaceted stroke-related problems can successfully lower the incidence of recurrent stroke and other cardiovascular events while also improving quality of life and functional outcome of patients with stroke [10]. Other studies are limited by relatively short follow-up periods; however, it is important to evaluate patient needs and benefits over longer periods [11–15]. Encouragingly, there is an enhanced focus on follow-up care of stroke survivors, as evidenced by a recent review on interventions provided to people with minor stroke. However, the review concluded that follow-up care mainly emphasizes secondary prevention rather than the wide range of other post-stroke consequences [16]. 

The aim of this longitudinal study was to evaluate a comprehensive and structured follow-up model over the first year after stroke using the Post-stroke Checklist (PSC). In a first phase of this study, we evaluated a structured follow-up at 3 months for patients with stroke, using a 14-item PSC to identify and intervene against stroke-related problems [17, 18]. In the present extension of the study, we evaluated the prevalence, cumulative number, and distribution of stroke-related health problems and their targeted healthcare interventions at 12 months after stroke. We also reported changes between 3 and 12 months and evaluated the longitudinal use of the PSC from a feasibility perspective.

## Methods

### Study population

The study population is described in a previous publication [17] and under ClinicalTrials.gov ID: NCT04295226. In short, all patients admitted to Skåne University hospital in Malmö, Sweden, for acute ischemic stroke (ICD-10 I.63) or intracerebral hemorrhage (ICD-10 I.61) and discharged directly to their own homes between February and April 2018 and June 2018 and February 2019 were invited to participate in the study. We excluded patients with dementia, severe comorbidity (severe psychiatric illness, kidney failure on dialysis, active cancer), or pre-stroke assisted living at the time of the index stroke. Home visits were not performed.

### Study design

This longitudinal explorative study included a face-to-face semi-structured nurse interview based on the PSC at 3 months and 12 months post-stroke, while also collecting information on risk factors, comorbidities, medications, and blood pressure. The overall purpose was to examine the feasibility of a comprehensive and structured follow-up program over time in stroke patients. We also recorded whether the PSC could be used in its entirety (*yes/no*), time used for screening each patient, and number of stroke-related health problems.

### The swedish 14-item post-stroke checklist

The Swedish modified PSC consists of 14 items with *yes/no* questions identifying patient-reported common stroke-related health problems. Beyond the 11 original items of the checklist, the 14-item version includes *fatigue*, *oral health and nutrition*, and *other challenges* related to stroke [18]. The 14 item PSC was developed from a pragmatic approach, the additional items were common reported problems suitable for intervention. The original 11 item PSC has been translated to Swedish and partly validated concerning feasibility and relevance [19]. To be defined as a stroke-related problem at 12 months, the problem had to be presumably linked to the index stroke, be covered by any of the 14 PSC items, and be new/persistent since 3-month follow-up, e.g., found it more difficult to take care of themselves (activities of daily living), communicating with others (communication), or had increased muscular stiffness (spasticity). At 12 months, items 2–4 in the PSC were defined as follows:

- *New problem*: patient did not report a problem within an item at 3-month follow-up, but experienced a new problem within the item at 12-month follow-up.
- *Persistent problem*: patient reported a problem within an item at 3-month follow-up, and the problem was persistent at 12-month follow-up.
- *Resolved problem*: patient reported a problem within an item at 3-month follow-up but did not report this at 12-month follow-up.

PSC items 2–14 were used in the same manner at 3 and 12 months using the questions “since your stroke…” and “since 3-month follow-up…”, respectively. However, PSC item 1 (secondary prevention) with the question “*Since your last visit*,* have you received any advice on health-related lifestyle changes or medications for preventing another stroke?*” was interpreted differently from items 2–14 at 12 months since absence of no advice since 3-month follow-up did not necessarily equal a problem. Therefore, item 1 in the PSC was defined as follows:

- *New problem*: need of an intervention related to secondary prevention at 12-month follow-up (*regardless* of the answer at 3-month follow-up).

### Multidisciplinary stroke team interventions

The nurse-led follow-up was followed by a multidisciplinary team conference where nurse(s), stroke physician(s), and occupational therapist(s) assessed the need of further interventions and tailored recommendations and advice. Other stroke team professions such as physiotherapists, speech therapists, welfare officers, or dietitians were consulted if required. The number of interventions and time used for team discussions and administering interventions were registered for each patient as part of the feasibility evaluation.

Interventions were primarily of two types: (1) additional patient-tailored advice and information and (2) referrals for rehabilitation, to general practice or to a specialist care clinic. Interventions made by doctors included referrals, changes in medication, and patient information. Interventions provided by other professions were information, tailored advice, referrals, and rehabilitation assessments. Interventions undertaken in the study were given in addition to standard care.

### Characteristics and follow-up data

Baseline characteristics including sex, age, pre-stroke living conditions, pre-stroke functional dependence (modified Rankin Scale (mRS) score 0–2 vs. 3–5), previous stroke or TIA, smoking status, secondary preventive medication prescribed at discharge, comorbidities diagnosed before or during hospitalization for stroke (hypertension, atrial fibrillation, diabetes), stroke subtype (ischemic stroke or intracerebral hemorrhage) were collected from the Swedish Stroke Register (Riksstroke), a nationwide hospital-based stroke register that covers > 90% of stroke patients admitted to hospital [20]. 

At the 12-month follow-up visit, we measured blood pressure, collected information about mRS, new stroke or transitory ischemic attack since index stroke, smoking status, and current secondary preventive medication.

### Statistics

Categorical variables were presented as proportions and quantitative variables as means or medians. Comparisons between groups were performed using the *X* [2] test for categorical variables and *t*-test for continuous variables. The association between mRS and stroke-related health problems was evaluated using the Kruskal–Wallis H test, and for comparing the mRS scores at 3 vs. 12 months we used the Wilcoxon signed-ranks test. The significance level was set to *p* ≤ 0.05 for all analyses. Statistical analyses were conducted using SPSS 26.0.

## Results

### Stroke survivors and patients lost to follow-up

**Fig. 1:**
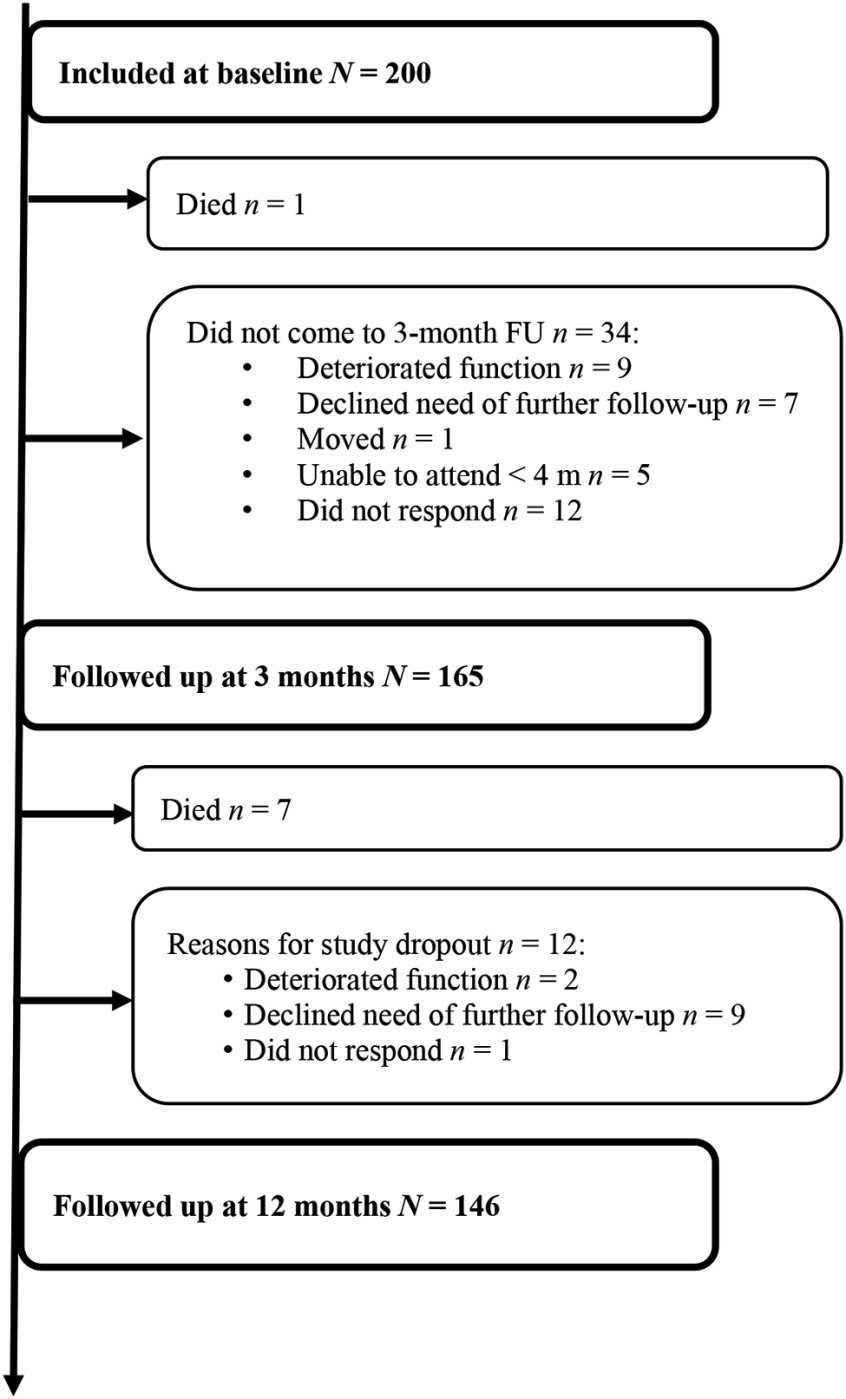
Study flowchart

We included a total of 200 patients at baseline. The follow-up rate at 12 months post-stroke was 77% (154/200): 8 patients died and 146 attended both 3- and 12-month follow-up visits. Reasons for loss to 12-month follow-up are shown in Fig. 1. There were no significant differences in age, sex, previous stroke status, stroke subtype, country of birth, or median number of reported stroke-related health problems at 3-month follow-up between those that were followed up and those who discontinued the study between 3 and 12 months.

### Demographics, comorbidity, recurrence, and secondary prevention

Data on patient demographics, comorbidities, and secondary prevention are presented in Table 1. The mean age at 12-month follow-up was 72 years (SD 12) and the proportion of women was 39%. Stroke recurrence between index stroke and 3-month evaluation was 1.4% (*n* = 2), whereas 6.2% (*n* = 9) had a recurrence beyond 3 months.

**Table 1:** Demographics, comorbidities, and secondary prevention in 146 patients followed up at 3 and 12 months post-stroke

Variable	Included patients *N* = 146			
	%		*n*	
**Demographics**				
Mean age (SD) 12-month FU	72 (12)			
Female sex	39%		57	
Single household	38.4%		53/138	
Pre-stroke living				
Own home without HCS	93.2%		136	
Own home with HCS	5.5%		8	
Other	1.4%		2	
Pre-stroke function				
Independent (mRS 0–2)	93.7%		134/143	
Dependent (mRS 3–5)	6.3%		9/143	
Highest education				
<9 years	32.2%		47	
10–12 years	34.9%		51	
>12 years	32.9%		48	
Country of birth				
Sweden	77.4%		113	
European	12.3%		18	
Non-European	10.3%		15	
Stroke subtype				
Ischemic	91.8%		134	
Hemorrhagic	8.2%		12	
**Vascular risk factors**				
Hypertension	78.1%		114	
Diabetes mellitus	24.0%		35	
Previous stroke	11.6%		17	
Previous TIA	7.5%		11	
Atrial fibrillation	24.7%		26	
Congestive heart failure	11.6%		17	
Coronary heart disease	15.2%		22/145	
Baseline smoking habit	20.5%		30	
**Other comorbidities**				
COPD	9.7%		14/145	
Chronic pain	17.8%		26	
Depression	8.9%		13	
Anxiety	2.7%		4	
Sleep disturbance	9.6%		14	
**Recurrence and secondary prevention**	**3 months**		**12 months**	
	**%**	**n**	**%**	**n**
Recurrent stroke after index stroke	1.4%	2	6.2%	9
Mean systolic BP (mmHg) (SD)	140 (20)		140 (20)	
Mean systolic MP (mmHg) (SD)	82 (12)		82 (12)	
Hypertension at FU (> 140 SBP / >90 DPB)	49%	71/145	42.8%	62/145
Antihypertensive treatment	82.9%	121	80.1%	117
Antiplatelet treatment (non-cardioembolic IS)	91.3%	94/103	88.3%	91/103
Statin treatment (all IS)	94.8%	127/134	87.3%	117/134
Anticoagulant treatment (AF and IS)	96.8%	30/31	87.1%	27/31
Current smoking habit (in smokers at baseline)	73.3%	22/30	76.7%	23/30
Smoking cessation (in smokers at baseline)	26.7%	8/30	23.3%	7/30

SD = standard deviation, HCS = Home care service, mRS = modified Rankin Scale, COPD = Chronic obstructive pulmonary disease,

FU = Follow-up, BP = blood pressure, AF = atrial fibrillation, IS = ischemic stroke, TIA = Transitory ischemic attack

Missing data: 5.5% for single household, ≤ 2% for all variables, the number of observations is stated under each carriable with missing data

### Functional outcome

**Fig. 2:**
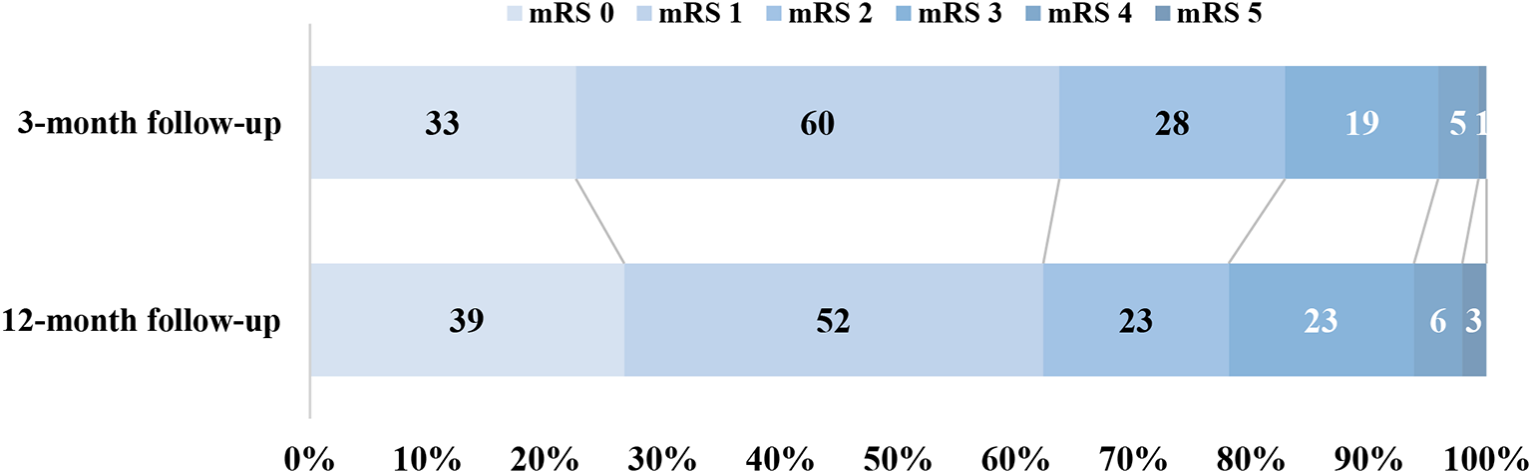
Functional outcome at 3-and 12-month follow-up assessed with the modified Rankin Scale, ranging from 0 to 5, with higher scores indicating more severe disability

Twelve months after the index stroke, 78% compared to 83% at 3 months, were functionally independent defined as a mRS score ≤ 2, see Fig. 2. We found no significant association between the median number of new stroke-related problems and level of dependency (dependent/independent) at 12 months, as opposed to the 3-month evaluation, where the median number of problems increased with increasing level of dependency. A total of 24% declined in functional status (higher mRS score) between 3 and 12 months, while 19% improved (lower mRS score) and 57% had an unchanged mRS score. Patients with worsened functional status were more likely to have had a new stroke (14.3%, *n* = 5 vs. 3.6%, *n* = 4, *p* = 0.022) than patients with improved or unchanged functional status based on the mRS score. Furthermore, they had higher prevalence of congestive heart failure (20% vs. 9.0%, *p* = 0.039), atrial fibrillation (37.1% vs. 20.7%, *p* = 0.049), and anxiety (8.6% vs. 0.9%, *p* = 0.015) (Supplemental Table 1).

### Changes in stroke-related health problems over the first year after stroke

**Fig. 3:**
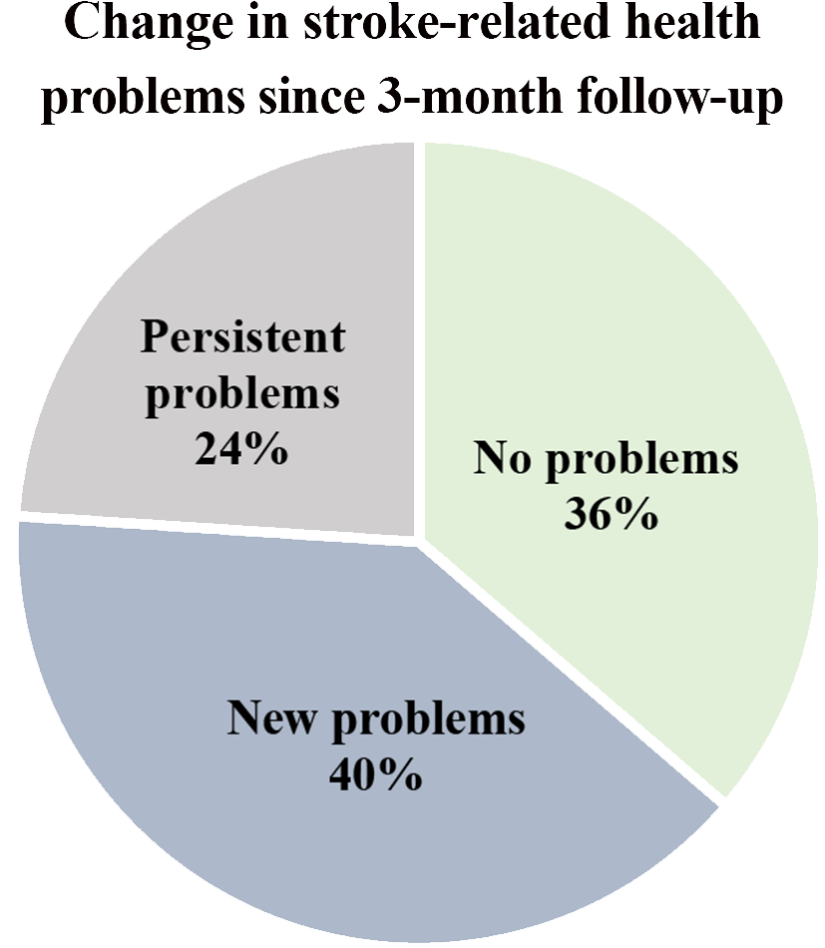
Pie chart presenting the change of stroke-related health problems since 3- month follow-up

Patients were divided into three groups based on their report of stroke-related health problems at 12 months: (1) new problems, (2) persistent problems and (3) no problems (including resolved problems), see Fig. 3. The prevalence of problems for every PSC item at 3 and 12 months is presented separately in Table 2. A more detailed presentation of the prevalence of problems can be seen in Supplemental Table 2.

**Table 2:** Stroke-related health problems identified using the Post-stroke Checklist at 3 and 12 months post-stroke

PSC item	3 months *N* = 146		12 months *N* = 146	
	%	*n*	%	*n*
1. **Secondary prevention** Unmet need of medical advice on health-related lifestyle changes or medications to prevent another stroke	*Have* *** not*** * received medical advice on health-related lifestyle changes or medications to prevent another stroke) since index stroke*		*Required* *** a new intervention*** * of any kind within secondary prevention*	
	57.5%	84	23.3%	34
	*Stroke-related health problem* (***new since index stroke***)		*Stroke-related health problem* (***new and persistent since 3 months***)	
	**%**	**n**	**%**	**n**
2. **ADL (activities of daily living)** Difficulties in ADL	22.6%	33	17.9%	26/145
3. **Nutrition** Oral health/nutrition problem	19.9%	29	12.5%	18/145
4. **Mobility** Difficulties walking or moving safely	31.5%	46	21.4%	31/145
5. **Spasticity** Increased muscular stiffness	8.2%	12	9.2%	12/145
6. **Pain** New pain	22.8%	33/145	15.7%	23
7. **Incontinence** Problems controlling bladder or bowel	17.1%	25	15.1%	22/145
8. **Communication** Difficulties communicating	26.7%	39	20%	29/145
9. **Mood** Anxiety or depressed mood	36.3%	53	24.9%	36/144
10. **Cognition** Difficulties to think, concentrate, or remember things	37.0%	54	30.2%	44
11. **Mental fatigue** Fatigue interfering with ability to do daily activities	47.3%	69	36.1%	52/144
12. **Life after stroke** Difficulties to carry out work, hobbies, sexuality, other activities, driving car	42.8%	62/145	33.4%	48/143
13. **Relationship with family** Difficulties in personal relationships	15.1%	22	10.2%	15
14. **Other challenges** Other challenges related to stroke	3.4%	5	2.1%	3

Missing data: ADL *n* = 1, nutrition *n* = 1, mobility *n* = 1, spasticity *n* = 1, incontinence *n* = 1, communication *n* = 1, mood *n* = 2, mental fatigue *n* = 2, life after stroke *n* = 3

#### Patients with new problems

The proportion of patients reporting any *new* problem at 12 months was 40% (58/146), compared to 90% (131/146) at 3 months. The highest proportion of new problems at 12 months was seen within *secondary prevention* (23%), followed by *life after stroke* (10%), *cognition* (6%), and *mood* (4.1%). No patient reported new problems within PSC item 14 – *other challenges related to stroke.*

**Fig. 4:**
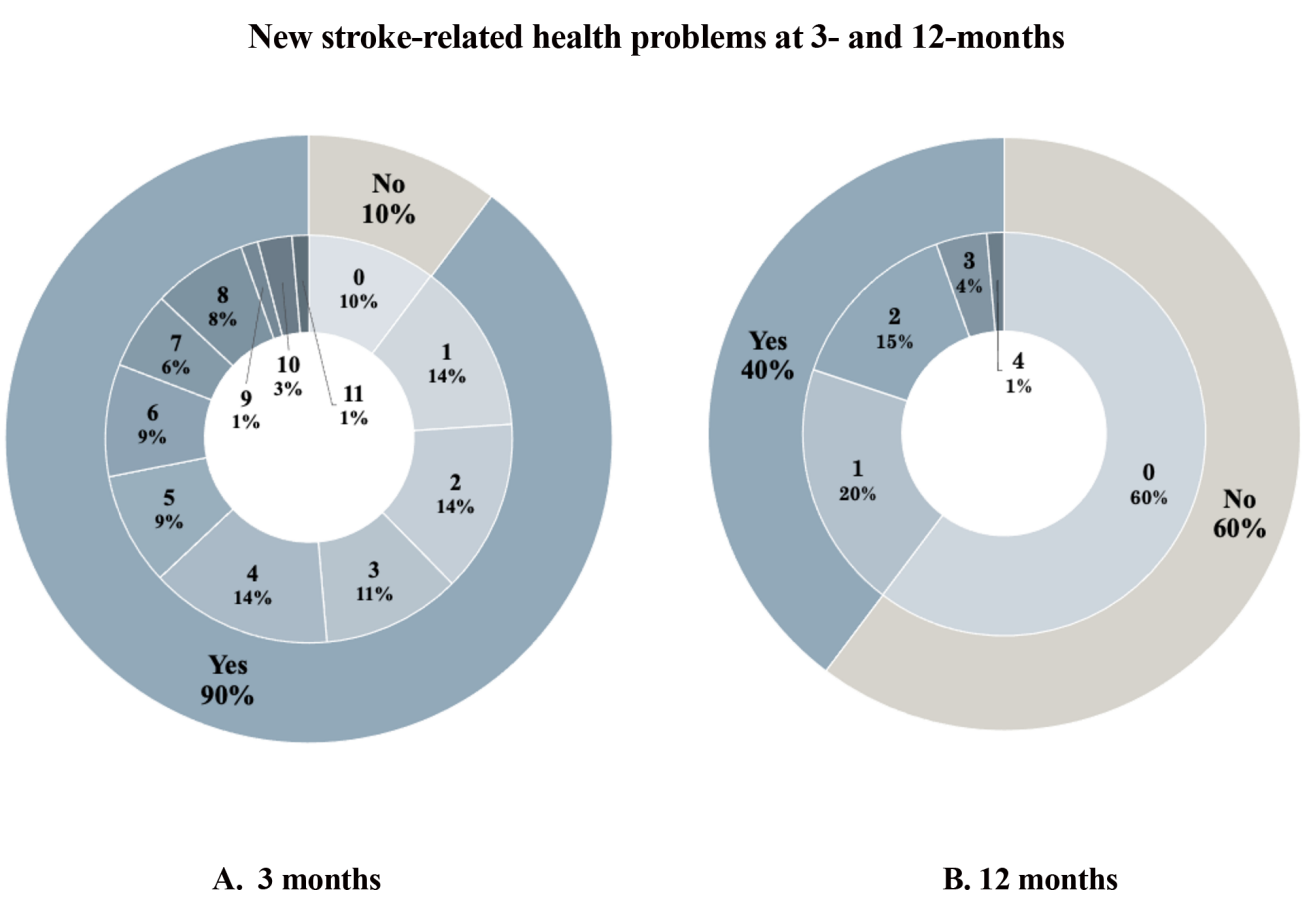
The outer layer presents the proportion of patients reporting new (since index stroke) problems at 3 months (**A**) and new (since 3-month evaluation) problems at 12 months (**B**), and the inner layer presents the cumulative number of new problems

The median number of new stroke-related health problems was zero per patient (IQR = 0–1) at 12 months and four (IQR = 2–6) at 3 months. Approximately one-third of patients (34%) reported 1–2 new problems, while only 6% reported three or more problems at 12-month follow-up. A comparison of new stroke-related health problems at 3 vs. 12 months post-stroke can be seen in Fig. 4.

Regarding PSC item 1 (secondary prevention) half of the patients (73/146) reported not having received advice on health-related lifestyle changes or medications to prevent new stroke since the 3-month follow-up. Approximately a fourth of patients (23%, 34/146) required an intervention of any kind within secondary prevention at 12-month follow-up, indicating a new problem within secondary prevention.

#### Patients with persistent problems

The proportion of patients that reported persistent but no new problems at 12-month follow-up was 24% (35/146). Persistent problems were most commonly reported within *fatigue* (33%), *cognition* (25%), *life after stroke* (23%), and *mood* (21%).

#### Patients with no problems

Approximately one third (36%, 53/146) of patients reported resolved problems or no new problems at 12 months, compared to 10% (15/146) at 3 months.

### Interventions for stroke-related health problems

**Fig. 5:**
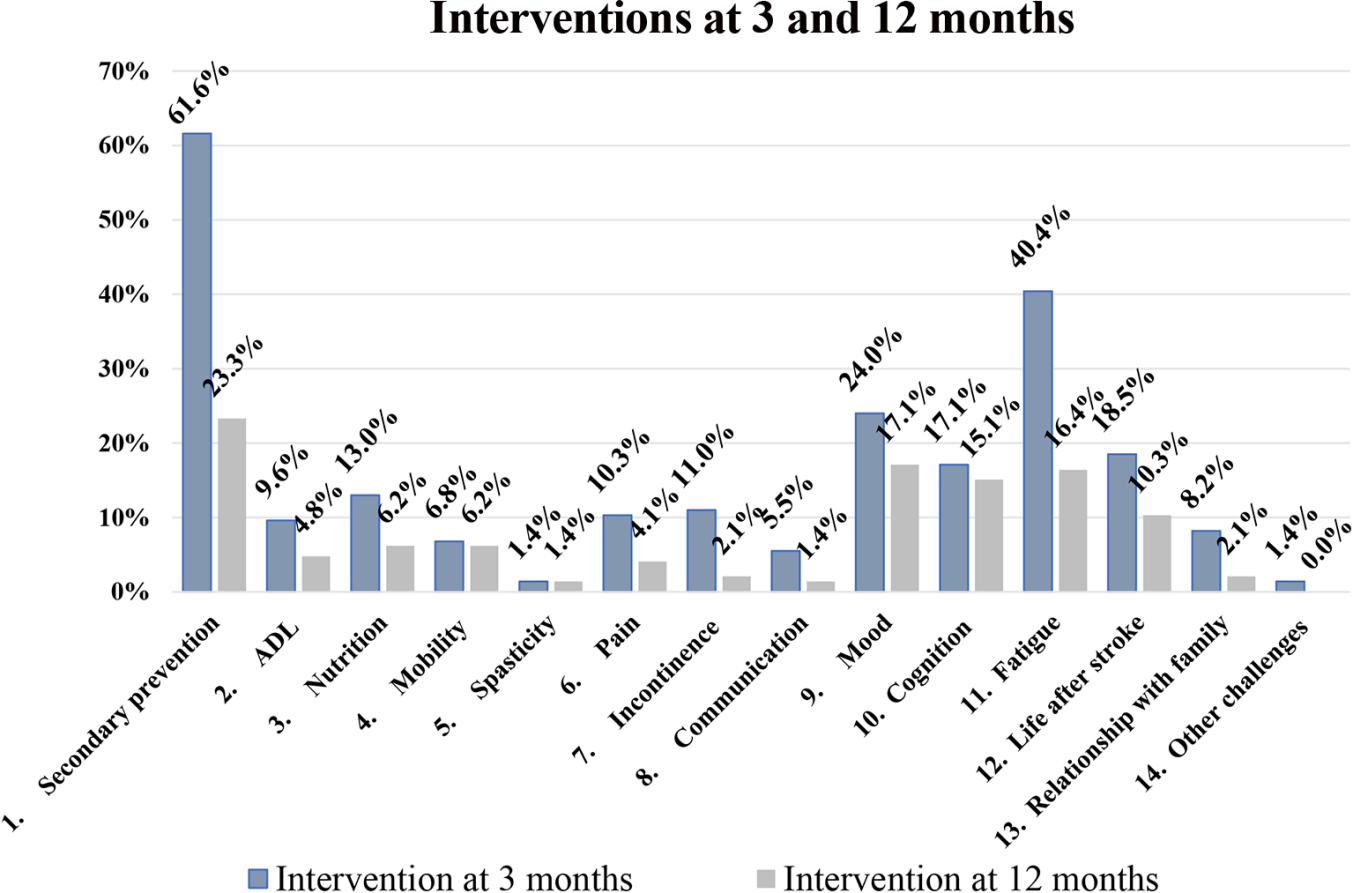
Proportions of patients in need of interventions in each of the 14 Post-Stroke Checklist items

Approximately half (53%, 78/146) of the patients received at least one new intervention at 12 months, compared to 84% (122/146) at 3 months. Specifically, in 49% (71/146) an intervention was prompted by a nurse or other stroke team professional and in 27% (39/146) by a physician. Interventions were mostly required within secondary prevention (23%), mood (17%), fatigue (16%), and cognition (15%), similar to interventions prompted at 3 months. Regarding secondary prevention, the most common areas for intervention were information/advice concerning stroke preventive measures given by a nurse (32%), information/advice concerning medications given by a nurse (21%), and primary care referral/information undertaken by a physician (50%). Interventions for mood, fatigue, cognition included tailored advice or referral to the Swedish Stroke Association/fatigue course, follow-up screening tools (HADS, MFS, MoCA) to detect a deterioration and referral to primary care/memory clinic/rehabilitation clinic. All new interventions for the individual PSC items at 3 and 12 months are presented in Fig. 5.

The median number of interventions per patient at 12 months was one (IQR = 0–2): one nurse or other stroke team professional intervention (IQR = 0–2) and zero physicians’ interventions (IQR = 0–1). It should be noted that not all identified stroke-related health problems generated new interventions since some patients already had ongoing interventions within routine healthcare. The most common type of intervention was information and tailored advice (49%), primary care referral (19%), and specialist care/rehab/other referral (8%).

### Feasibility evaluation

The semi-structured PSC interview containing 14 items could be completed in its entirety in all 146 patients. The median time to complete the PSC interview at 12 months was 28 min (IQR 18.5–40, range: 6–100) compared to 30 min (IQR = 22–45, range: 5–140) at 3 months.

## Discussion

### Summary of findings

We found that one-third of the patients had completely recovered, reporting no stroke-related health problems during the 12-month evaluation according to the PSC. However, the remaining two-thirds experienced persistent or new problems related to their strokes. Notably, the proportion reporting new problems was 40% at 12 months, compared to the 90% at 3 months. The most commonly reported new problems were within the domains of *secondary prevention*,* life after stroke*, and *cognition.* Additionally, at 12 months, half of the patients needed new healthcare interventions, compared to 86% at 3 months. Despite the reduction in stroke-related health problems and their targeted interventions between the 3- and 12-month evaluations, problems were experienced by two-thirds of the patients at 1 year, emphasizing the relevance of continuous follow-up of stroke survivors.

From a feasibility perspective, the PSC was completed in all patients at 12 months with a median time of 28 min. PSC item 1 (*secondary prevention*) had to be modified for the 12-month follow-up, whereas the rest of the PSC items (2–14) could be used without change at the two timepoints. Item 14 (*other challenges related to stroke*) was not reported by any patient at 12 months and by 1.4% at 3 months. Although our findings confirm the comprehensiveness of items 1–13, the continued clinical use of item 14 could be questioned. Most interventions could be carried out by a nurse, supporting the overall feasibility of the nurse-based model. This study evaluated a hospital-based follow up handled by trained nurses with competence on stroke. The generalizability to use of the model primary care where stroke patients are fewer, may therefore be limited.

The proportion of women in the study was lower (39%) than in the general stroke population which has consistently been 46% in the Swedish Stroke Register. Women experience stroke later in life and were less often discharged straight to their own homes, and therefore not eligible for the study. This at least in part explains the observed difference [21]. 

We observed a functional decline in a fourth of the patients between 3 and 12 months, predominantly in patients with recurrent stroke or significant comorbidity. On the other hand, the functional status improved in approximately one-fifth of patients. No significant association between the median number of new stroke-related problems and the level of dependency at 12 months was found. Of note, 64% of patients experienced new or persistent stroke-related problems, while 74% remained functionally independent (mRS ≤ 2) at 12 months, thereby showing that stroke-related health problems are poorly reflected by the mRS. It has previously been shown that patients with a favorable mRS outcome often experience cognitive impairment, difficulties with social reintegration, and depression [22]. 

### Our research in context of current knowledge

The PSC has been validated in several studies, supporting its feasibility in different settings [11, 12, 14]. A study investigating the prevalence of worsening problems using the PSC at 3, 6, and 12 months post-stroke found that mood disturbances were the most frequently and continuously identified worsened problem and that PSC was useful for the detection of worsened problems [15]. A cross-sectional study comparing stroke-related health problems using the PSC across seven countries at 6 months post-stroke implied that the most prevalent problems were cognition, life after stroke, and mood [13]. These results are in line with our findings that stroke-related health problems are often persistent and even worsened beyond the sub-acute phase and are particularly common within the non-motor symptoms of stroke.

Long-term risk factor control and adherence to recommended medications and guidelines are often suboptimal in routine healthcare [23–26]. Reasons for lack of adherence include insufficient monitoring or treatment modifications/intensification when therapeutic response is not obtained, but also include patients making decisions about medications independently of their general practitioner, or prioritizing other aspects like quality of life rather than striving for treatment targets [27–30]. Despite our targeted interventions regarding secondary prevention, results were discouraging. The reduction in patients presenting with high blood pressure from 49% at 3 months to 43% at 12 months was modest at best. 80% of all patients were on antihypertensives at 12 months, implying that dose titration or intensification of treatment may be important challenges. Statin use was discontinued by 8% of patients between the two follow-ups, and 77% of baseline smokers still smoked at 12 months. Our intervention, which was an add-on to routine healthcare, did not include a doctor’s visit and did not have resources for continuous contact with the patient but served as a tool to identify problems. Thus, we had to rely on primary care to reach secondary prevention treatment targets. The rate of stroke recurrence was 7.5%. Some of these strokes might have been prevented by improved risk factor control. We acknowledge that use of our model alone for follow-up is insufficient for reaching long-term treatment targets. Primary care differs between countries, but patients are severely underserved in Sweden, which may affect patients with chronic diseases and regular follow-up needs. It could be argued that patients with stroke are best managed at a stroke clinic during the first important year post-stroke. In conclusion, the long-term care of stroke patients and strategies for reaching secondary prevention targets warrant further studies. To align with the targets for stroke 2030 in the Stroke Action Plan for Europe, stating that at least 90% of stroke patients are to be seen by a stroke specialist, have access to secondary prevention and to include secondary prevention in the national stroke plans with follow up in primary care. Currently, no European country has a fully implemented long term care program for stroke.

### Strengths and limitations

The strengths of this study include a consecutive cohort of patients with detailed data and a longitudinal design, allowing for analyses of changes over time. The study has numerous limitations. Firstly, the lack of a control group provides limited ability to analyze the effectiveness of our model compared to standard of care. An additional limitation is that follow-up was limited to patients that were discharged home and excluded patients with severe comorbidity or dementia. This study was performed in a clinical setting (stroke-unit) and would mimic a normal clinical situation; hence we excluded patients not able to visit the out-patient clinic. It should be noted that this study is part of a larger study that also evaluates stroke patients discharged to nursing homes. However, it should be taken into account that 75% of stroke patients in Sweden are discharged to home [8]. As home discharge represents the majority within the stroke population it is seemingly relevant to conduct a study on this population. Another study assessing patients with severe strokes that were discharged to nursing homes found the PSC to be useful in that patient group [31]. Interestingly, the nature of stroke-related health problems differed markedly between patients discharged home and those discharged to nursing homes and ought to be studied separately. The follow-up rate was 77%. Patients lost to follow-up at 3 months were older, and the most common reason for study exclusion was declined function (9/34). Twelve patients were lost between 3 and 12 months, and the most common reason for discontinuing the study was declined need for continued follow-up. Home visits were not offered but might have prevented drop-out due to decline in function.

Single blood pressure measurements, which are known to be less precise than 24-hour measurements, were used at 3 and 12 months and may have overestimated the prevalence of hypertension.

## Conclusions

Stroke-related health problems decreased over time but were still experienced by two-thirds of patients 1 year after the stroke. Continuous stroke follow-up therefore remains highly relevant. The nurse-based follow-up model with stroke team support used in this study was feasible, but its use alone is insufficient to reach secondary prevention targets. With few modifications, we found the PSC to be feasible for longitudinal use and to capture stroke-related health problems during the first year. Further development of PSC-based follow-up models could focus on sustainable and intensified secondary prevention strategies.

## Electronic supplementary material

Below is the link to the electronic supplementary material.

Supplementary Material 1

Supplementary Material 2

## Data Availability

Requests to access an anonymized dataset supporting the conclusions of this article may be sent to the corresponding author after obtaining the appropriate ethics approval.

## Declarations

### Guarantor

KM.

## Abbreviations

PCS: Post stroke checklist
SSNAPP: The Sentinel Stroke National Audit Programme
ICD: International Statistical Classification of Diseases and Related Health Problems
mRS: Modified Rankin Score
IQR: Interquartile range
