# Supplementary material for: The Post-Stroke Checklist: longitudinal use in routine clinical practice during first year after stroke
Source: BMC Cardiovasc Disord. 2024 Oct 29;24:601. doi: 10.1186/s12872-024-04239-6 (PMC11520836; doi:10.1186/s12872-024-04239-6)
Supplement: Supplementary file 1 — Supplementary Material 1 [file 12872_2024_4239_MOESM1_ESM.docx]

| **Supplemental Table 1.** *Characteristics of study population by change of functional outcome (mRS) between 3 and 12 months.* | | | |
| --- | --- | --- | --- |
| **Characteristics** | **Declined functional status**  **(*n* = 35)** | **Unchanged or improved functional status *(n* = 111)** | ***p-*value** |
| Age, mean (SD) | 73 (12) | 72 (12) | 0.583 |
| Female sex | 38.2% | 38.7% | 0.958 |
| Recurrent stroke^*^ | 14.3% | 3.6% | 0.022 |
| Stroke subtype  Ischemic stroke  Intracerebral hemorrhage | 88.6%  11.4% | 92.8%  7.2% | 0.428  0.428 |
| Hypertension | 68.6% | 81.1% | 0.119 |
| Atrial fibrillation^*^ | 37.1% | 20.7% | 0.049 |
| Previous stroke | 14.3% | 10.8% | 0.576 |
| Diabetes | 28.6% | 22.5% | 0.465 |
| COPD | 8.6% | 10.0% | 0.803 |
| Congestive heart failure^*^ | 20.0% | 9.0% | 0.039 |
| Coronary heart disease | 8.6% | 17.3% | 0.211 |
| Chronic pain | 20.0% | 17.1% | 0.697 |
| Depression | 8.6% | 9.0% | 0.937 |
| Anxiety^*^ | 8.6% | 0.9% | 0.015 |
| A significant difference (*p* < 0.05) between groups is marked with ^*^. | | | |
