# Supplementary material for: The Post-Stroke Checklist: longitudinal use in routine clinical practice during first year after stroke
Source: BMC Cardiovasc Disord. 2024 Oct 29;24:601. doi: 10.1186/s12872-024-04239-6 (PMC11520836; doi:10.1186/s12872-024-04239-6)
Supplement: Supplementary file 2 — Supplementary Material 2 [file 12872_2024_4239_MOESM2_ESM.docx]

| **Supplemental Table 2*.*** *Stroke-related health problems identified using the Post-Stroke Checklist at 3 and 12 months post-stroke.* | | | | |
| --- | --- | --- | --- | --- |
| **PSC item** | **3 months N = 146** | | **12 months N = 146** | |
|  | **%** | **n** | **%** | **n** |
| 1. **Secondary prevention**   Unmet need of medical advice on health-related lifestyle changes or medications to prevent another stroke | *Did* ***not*** *received medical advice on health-related lifestyle changes or medications to prevent another stroke) since index stroke* | | *Required* ***a new*** ***intervention*** *of any kind within secondary prevention* | |
|  | 57.5% | 84 | 23.3% | 34 |
|  | *Stroke-related health problem*  *(****new since index stroke****)* | | *Stroke-related health problem*  *(****in relation to problems at 3 months****)* | |
|  | **%** | **n** | **%** | **n** |
| 1. **ADL (activities of daily living)**   Difficulties in ADL | 22.6% | 33 | New: 2.1%  Persistent: 15.8%  – *increased*: 0.7%  – *improved:* 7.5%  – *unchanged:* 7.5%  Resolved: 6.3% | 3/145  23/145  1/145  11/145  11/145  9/145 |
| 1. **Nutrition**   Oral health/nutrition problem | 19.9% | 29 | New: 2.8%  Persistent: 9.7%  - *increased*: 2.9%  - *improved:* 2.1%  - *unchanged*: 4.8%  Resolved: 10.3% | 4/145  14/145  4/145  3/145  7/145  15/145 |
| 1. **Mobility**   Difficulties walking or moving safely | 31.5% | 46 | New: 2.1%  Persistent: 19.3%  - *increased*: 4.1%  - *improved:* 9.6%  - *unchanged*: 5.5%  Resolved: 11.7% | 3/145  28/145  6/145  14/145  8/145  17/145 |
| 1. **Spasticity**   Increased muscular stiffness | 8.2% | 12 | New: 3.4%  Persistent: 4.8%  - *increased*: 0%  - *improved:* 3.4%  - *unchanged*: 1.4%  Resolved: 2.8% | 5/145  7/145  0/145  5/145  2/145  4/145 |
| 1. **Pain**   New pain | 22.8% | 33/145 | New: 2.7%  Persistent: 13%  - *increased*: 2.7%  - *improved:* 6.2%  - *unchanged*: 4.1%  Resolved: 9.6% | 4  19  4  9  6  14 |
| 1. **Incontinence**   Problems controlling bladder or bowel | 17.1% | 25 | New: 1.4%  Persistent: 13.7%  *- increased*: 1.4%  - *improved:* 3.4%  - *unchanged*: 8.9%  Resolved: 2.8% | 2/145  20/145  2/145  5/145  13/145  4/145 |
| 1. **Communication**   Difficulties communicating | 26.7% | 39 | New: 2.1%  Persistent: 17.9%  - *increased*: 2.1%  - *improved:*12.3%  - *unchanged*: 3.4%  Resolved: 8.3% | 3/145  26/145  3/145  18/145  5/145  12/145 |
| 1. **Mood**   Anxiety or depressed mood | 36.3% | 53 | New: 4.1%  Persistent: 20.8%  - *increased*: 5.6%  - *improved:*8.2%  - *unchanged*: 6.8%  Resolved: 15.8% | 6/144  30/144  8/144  12/144  10/144  23/144 |
| 1. **Cognition**   Difficulties to think, concentrate, or remember things | 37.0% | 54 | New: 5.5%  Persistent: 24.7%  - *increased*: 6.8%  - *improved:*8.2%  - *unchanged*: 9.6%  Resolved: 12.3% | 8  36  10  12  14  18 |
| 1. **Mental fatigue**   Fatigue interfering with ability to do daily activities | 47.3% | 69 | New: 3.5%  Persistent: 32.6%  - *increased*: 2.8%  - *improved:* 22.6%  - *unchanged*: 6.8%  Resolved: 14.5% | 5/144  47/144  4/144  33/144  10/144  21/144 |
| 1. **Life after stroke**   Difficulties to carry out work, hobbies, sexuality, other activities, driving car | 42.8% | 62/145 | New: 10.3%  Persistent: 23.1%  - *increased*: 4.2%  - *improved:* 9.6%  - *unchanged*: 8.9%  Resolved: 18.2% | 15/143  33/143  6/143  14/143  13/143  26/143 |
| 1. **Relationship with family**   Difficulties in personal relationships | 15.1% | 22 | New: 3.4%  Persistent: 6.8%  - *increased*: 1.4%  - *improved:* 3.4%  - *unchanged*: 2.1%  Resolved: 8.2% | 5  10  2  5  3  12 |
| 1. **Other challenges**   Other challenges related to stroke | 3.4% | 5 | New: 0%  Persistent: 2.1%  - *increased*: 0%  - *improved:*1.4%  - *unchanged*: 0.7%  Resolved: 1.4% | 0  3  0  2  1  2 |
| Missing data: ADL *n* = 1, nutrition *n* = 1, mobility *n* = 1, spasticity *n* = 1, incontinence *n* = 1, communication *n* = 1, mood *n* = 2, mental fatigue *n* = 2, life after stroke *n* = 3 | | | | |
